# Factors determining patient satisfaction during healthcare delivery in Limpopo, South Africa

**DOI:** 10.4102/curationis.v49i1.2819

**Published:** 2026-03-19

**Authors:** Marubini Y. Rambuwani, Lebogang L. Molefe, Lucky O. Letswalo

**Affiliations:** 1 Department of Nursing, Faculty of Health Sciences, Sefako Makgatho University, Pretoria, South Africa

**Keywords:** determinants, delivery, factors, healthcare, patient, satisfaction

## Abstract

**Background:**

Patient satisfaction is a key indicator of healthcare quality. Several studies conducted revealed that there is patient dissatisfaction globally, in the sub-Saharan region and nationally. However, factors influencing patients’ satisfaction during healthcare delivery remain unknown.

**Objectives:**

The study aimed to examine the factors that influence patients’ satisfaction during healthcare delivery at a selected district hospital.

**Method:**

A quantitative descriptive design was used. The questionnaire was developed using the Donabedian model for quality care to measure patient satisfaction. The tool was piloted before use. Questionnaires were distributed to 320 respondents who were randomly selected. The response rate was 100%. Data were analysed using statistical software, SAS 9.2, with descriptive statistics and the Chi-square tests.

**Results:**

Key issues that made patients dissatisfied with healthcare facilities were poor communication, inpatient disagreement (19% as compared to 6% outpatient, *p* = 0.0018), long waiting time (76% of inpatients as compared to 24% of outpatient respondents, *p* < 0.001), unclean environment (26% inpatient and 35% outpatient), dirty linen, unsafe water for drinking, and poor infrastructure.

**Conclusion:**

It is evident that many issues contribute to patient dissatisfaction in healthcare facilities, highlighting the need for improvement. Satisfied patients are more likely to return, while dissatisfied ones may neglect treatment, spread negative feedback, or pursue legal action. Such dissatisfaction can also discourage others from seeking care. Therefore, healthcare providers must regularly assess their services, identify areas for improvement, and implement changes to maintain patient satisfaction. Furthermore, recommendations were made on how to improve the healthcare facilities. Implementing the recommended strategies can greatly enhance quality care and patient satisfaction.

**Contribution:**

The findings will contribute to the development of quality improvement strategies and monitoring systems.

## Introduction

Patient satisfaction is central to healthcare delivery. Admission to a healthcare facility often separates patients from the comfort and support of home, making it vital to provide a warm, comfortable, and therapeutic environment that prevents loneliness, homelessness, and regret. Patient comfort, defined as the sense of happiness, relaxation, and satisfaction during care, positively influences well-being, shapes perceptions of the care process, and leads to faster recovery and better outcomes (Tian 2023). Quality care, therefore, is essential to promote patient satisfaction. It involves holistic and competent care that addresses all patient needs and aims for optimal results, supported by effective communication, teamwork, leadership, personal commitment, safety, person-centred care, staff competence, and patient participation in decision-making (Stavropoulou et al. 2022:472).

The World Health Organization (WHO) estimates that two billion people live in fragile, conflict-affected, and vulnerable settings, making it essential to ensure quality healthcare services for all (WHO 2020). Poor quality care remains a global concern, contributing to patient safety incidents, hospital-acquired infections, high mortality rates, and excessive healthcare costs (Endalamaw et al. 2023:893). Medical errors cost an estimated USD 42 billion annually, and 1 in 10 patients in high-income countries experiences errors during hospital care. In developing countries, 7 in 100 hospitalised patients acquire healthcare-associated infections. In 137 low- and middle-income countries, poor quality care causes approximately 5 million deaths annually, resulting in USD 1.4 to 1.6 trillion in lost productivity (Endalamaw et al. 2023:893).

In Africa, a study by Sinyiza et al. (2022) found that patients’ satisfaction with healthcare facilities remains a challenge. Patients raised six major issues that dampened their healthcare-seeking experience, including health workers reporting late to work, doctors not listening to patients’ concerns and neither examining them properly nor explaining the diagnosis, shortage of medicines, diagnostics and medical equipment, unprofessional conduct of health workers, poor sanitation and cleanliness, and health worker behaviour of favouring relatives and friends over other patients. Another study by Mwebesa, Kayemba and Mutanda (2025) revealed that patients are not satisfied with the physical environment within the healthcare facilities.

South Africa is a developing country among the above-mentioned countries, where quality care remains an issue. Tenza et al. (2024:324) conducted a study in all nine provinces of the country on the quality of care and patient safety. The study revealed that there is poor quality of care and poor patient safety in the country. This occurs despite the existence of policy documents that govern the quality of care and patient safety, policies such as the Patient Rights Charter (National Department of Health [NDoH] 2016), the Batho Pele principles (Department of Public Service and Administration 2014), and the national guideline for patient safety incident reporting and learning in the public health sector (NDoH 2011). In response, the government is strengthening the health system through initiatives such as the Presidential Health Compact 2024–2029, signed on 22 August 2024, and the *National Health Insurance (NHI) Act*, which aims to ensure equitable, effective, and resource-efficient healthcare for all (NHI 2023).

Limpopo province is one of the nine provinces of South Africa. Quality of care remains inconsistent in the province. A study by Nyelisani, Makhado and Luhalima (2023) revealed that there is a lack of equipment, inadequate training, limited infrastructure, challenges relating to non-adherence to protocols and instructions in Limpopo province, making it difficult to provide optimal quality care. It is against this background that the study was prompted. Therefore, the study aims to examine the factors that influence patients’ satisfaction during healthcare delivery at a selected district hospital.

### Purpose of the study

The study aims to examine the factors that influence patients’ satisfaction during healthcare delivery at a selected district hospital.

## Research methods and design

### Study design

A quantitative descriptive design was used. This design is suitable for the phenomenon under study as it allows the researcher to collect numerical data from many patients and describe their satisfaction levels accurately. This design seeks to identify factors – such as waiting time, communication, and staff attitude – that influence satisfaction. Using structured surveys, it ensures consistent, objective, and reliable data for clearly describing patient experiences. Moreover, it is a method that collects and analyses numerical data to describe the characteristics of a population or phenomenon under study, and has clearly defined research questions (Slater & Hasson 2024).

### Setting

The study was conducted in Limpopo province, South Africa. The selected district hospital was also appropriate because it is a busy 122-bed facility that sees approximately 1200 outpatients monthly and admits around 400 inpatients in various wards per month, including medical, surgical, antenatal, and postnatal wards, and also provides operative and emergency care services. It has a large patient size and a diverse case mix, providing a rich environment for gathering reliable information on patient satisfaction.

### Study population and sampling strategy

The study population comprised all hospital patients, both outpatients and inpatients. Systematic random sampling was applied by calculating the sampling interval (*k*) using the formula: *k* = N.n.

In the formula, N is the population (number of patients in the ward, e.g. 35), and n is the targeted sample size (8) of the ward per day (Polit & Beck 2018:289). If the sampling interval (*k*) is 8, and the sampling started by randomly selecting the patient’s bed number 3, then applying the sampling interval, the third number was 11, followed by the number 19 (Polit & Beck 2018:748). A two-stage process was used: firstly, patients were stratified into inpatients and outpatients. Eight inpatients were to be selected daily for 20 days. Secondly, eight outpatients were to be randomly selected for 20 days. Therefore, the total number of both inpatients and outpatients was 160 each, totalling 320.

A multi-stage sampling method further divided groups into smaller units, enabling detailed examination of population structures (Okechi 2024).

### Inclusion and exclusion criteria

The inclusion criteria were all inpatients who were admitted to the hospital, plus all outpatients who visited the hospital to seek healthcare. Furthermore, patients were older than 18 years to give voluntary consent to participate and were well-oriented and aware. Patients in the surgical and medical wards were given equal opportunities, but only those who could make informed decisions were allowed to participate.

All patients seen in the emergency room and operating room were excluded because of the emergency nature of the care they had to receive. Furthermore, all patients under 18 years of age and those who were mentally unstable were excluded.

### Data collection instrument and procedure

A structured questionnaire was developed using the Donabedian model for quality care (2005) to measure patient satisfaction with healthcare. This questionnaire was developed with the assistance of a statistician and had 32 questions grouped into four sections. Section A covered demographic information, Section B assessed satisfaction with care received, Section C examined satisfaction with the quality of care provided, and Section D contained three open-ended questions for additional views and suggestions. The views and experiences of the respondents were considered. The tool was piloted with 16 randomly selected inpatients and 16 outpatients to assess feasibility and practicality. Therefore, 32 patients participated in the piloting process. During the pilot, the inpatients completed the questionnaire in a side room, while the outpatients used the pharmacy boardroom at their exit point. During actual data collection, the same tool that was piloted was used with minor amendments, and the data from the pilot study was not part of the main study, as there was a need for minor amendments to the data collection tool. The questionnaires were distributed to 320 respondents. Eight inpatients were to be selected daily for 20 days from a random starting point as per the total number of patients on that specific day during data collection. Furthermore, eight outpatients were to be randomly selected for 20 days so that each patient had an equal opportunity. Therefore, the total number of both inpatients and outpatients was 160 each, totalling 320, who were randomly selected. A total of 240 (*n* = 240) were outpatients, and 80 (*n* = 80) were inpatients. All respondents completed the questionnaires and returned them to the investigator. The study was carried out face-to-face in a side room and in a pharmacy boardroom.

Respondents who could not read and write were assisted in completing the questionnaire. The response rate was therefore 100%.

### Data analysis

Data were analysed using statistical software, SAS 9.2, with descriptive statistics and Chi-square tests. The data were described using the Chi-square test (χ^2^) for the equal proportion technique.

The results were presented in tables, graphs, and bar charts. The responses for open-ended questionnaires were analysed by the researcher, using sentiment data analysis that assisted in grouping and categorising sentiment. The sentiment was confirmed by the statistician. The findings were presented in the report using bar charts.

### Validity and reliability

Rigour was ensured through validity and reliability, with assistance from a statistician, to accurately assess patient care at the selected hospital (Polit & Beck 2021:690). For this study, face validity was established through pre-testing to confirm that the questions were relevant, clear, and easy to understand. Content validity was ensured by including sufficient content to measure the phenomenon under study, supported by Polit and Beck (2021:690). Additionally, the researcher ensured that the questions were formulated to elicit the variables that influence the care of the patient at the selected hospital. Adjustments to the questionnaires were made after pilot study discussions with the statistician. Reliability is the accuracy and consistency of information (Polit & Beck 2021:691), and this was achieved by ensuring that the information was obtained directly from the respondents.

### Ethical considerations

Ethical clearance to conduct this study was obtained from the University of Pretoria Faculty of Health Sciences Research Ethics Committee with the ethical approval number 406/2017 and the Provincial Department of Health Ethics Committee with ethical approval number 4/2/2. A letter was received from the CEO of the hospital who granted permission for the study to be conducted there. Informed consent was obtained before data collection. Respondents were assured of their rights to withdraw at any time should they feel like, the right to be protected from harm, the right to confidentiality, and privacy.

## Results

### Response rate

Respondents were male and female participants of various age groups and educational levels. All 320 respondents responded, comprising 160 inpatients and 160 outpatients. The response rate was therefore 100%. All questionnaires were completed appropriately and returned to the investigator.

### Demographic characteristics

Table 1 presents the respondents’ demographic characteristics, including age, gender, educational level, hospital visits, previous admissions, and wards of admission. A Chi-square test assessed the relationships between demographics and determinants of patient satisfaction. The sample included both male and female participants, of various age groups, and respondents with varying educational levels, including some with no formal education.

**TABLE 1:** Respondents’ demographic characteristics (*N* = 320).

Demographic	Frequency	%	Chi-square	Probability
**Gender**				
Male	105	33	37.81	< 0.001
Female	215	67	-	-
**Age group (years)**				
≤ 35	138	43	60.68	< 0.001
36–60	141	44	-	-
> 60	41	13	-	-
**Level of education**				
No School	26	8	212.92	< 0.001
Primary	37	12	-	-
Secondary	190	59	-	-
Tertiary	67	21	-	-

*Source*: Adapted from Donabedian, A., 2005, ‘Evaluating the quality of medical care’, *Milbank Quarterly* 83(4), 691–729. https://doi.org/10.1111/j.1468-0009.2005.00397.x

Table 2 presents the patient visit patterns. There was a highly significant difference (*p* < 0.001) between inpatients and outpatients. Eighty-four inpatients reported occasional visits, compared to 40 outpatients, while 59 outpatients visited monthly, versus 15 inpatients, mainly to collect scheduled chronic medication. Over 50 respondents in each group reported never being admitted. There was a significant association between outpatients (68%) agreeing that nurses sit and talk to patients and inpatients (49%) (*p* = 0.0065, *χ*^2^ = 10.09). Similarly, inpatients showed higher disagreement (19%) with nurses explaining their condition compared to outpatients (6%) (*p* = 0.0018, *χ*^2^ = 12.68). Inpatients also reported a higher disagreement (56%) regarding information on other available services compared to dissatisfaction with other factors.

**TABLE 2:** Patients’ visit patterns.

Hospital visits	Inpatients	Outpatients	Chi-square	Probability
Monthly	15	59	-	-
Occasionally	84	40	51.47	< 0.001
Weekly	0	2	-	-
Yearly	1	0	-	-

*Source*: Adapted from Donabedian, A., 2005, ‘Evaluating the quality of medical care’, *Milbank Quarterly* 83(4), 691–729. https://doi.org/10.1111/j.1468-0009.2005.00397.x

Table 3 shows the relationship between patients’ categories (inpatients and outpatients) and communication, as well as how patients responded regarding communication. A significant relationship was found between outpatient agreement (68%) and inpatient agreement (49%) on how nurses sit and talk to patients (*p* = 0.0065, *χ*^2^ = 10.09). Another significant relationship was observed between inpatient disagreement (19%) and outpatient disagreement (6%), concerning nurses explaining their condition, as well as the failure of healthcare providers to provide information on other services (*p* = 0.0018, *χ*^2^ = 12.68).

**TABLE 3:** How patients responded regarding communication.

Communication	Inpatients	Outpatients	Chi-square	Probability
**Nurses use understandable language**			0.94	0.6258
Agree	98	98	-	-
Neutral	59	61	-	-
Disagree	3	1	-	-
**I am involved in my health decisions**			0.34	0.8417
Agree	78	75	-	-
Neutral	66	66	-	-
Disagree	16	19	-	-
**Given the service available information**			5.34	0.0693
Agree	38	52	-	-
Neutral	64	63	-	-
Disagree	58	45	-	-
**Given health education**			3.43	0.1791
Agree	73	78	-	-
Neutral	59	62	-	-
Disagree	28	20	-	-
**The nurse sits and talks to me**			10.09	0.0065
Agree	49	68	-	-
Neutral	62	62	-	-
Disagree	49	30	-	-
**The nurse explains what is wrong with me**			12.68	0.0018
Agree	81	91	-	-
Neutral	60	63	-	-
Disagree	19	6	-	-

*Source*: Adapted from Donabedian, A., 2005, ‘Evaluating the quality of medical care’, *Milbank Quarterly* 83(4), 691–729. https://doi.org/10.1111/j.1468-0009.2005.00397.x

Table 4 presents patients’ experience of care with the general service. The waiting time was strongly associated with inpatient responses (*p* < 0.001). Dissatisfaction with long waiting times to see a doctor was reported by 76% of inpatients compared to 24% of outpatients. Over half of the outpatients felt the wait was adequate, while only 16% of inpatients agreed. Overall, long waiting times were a key factor in dissatisfaction, with inpatients reporting the highest levels. Patients reported high satisfaction with cleanliness (*p* = 0.0459) and a clean environment (*p* = 0.0382), with nursing care scores above 80%. Most agreed on the positive quality of care, while only a few disagreed or remained neutral. The majority (90%) were pleased with the hospital’s care, and over 80% agreed the reception during admission was excellent, with friendly and helpful staff. Although agreement levels were high, the difference between patient categories was not statistically significant (*p* > 0.05). Most respondents (56%) expressed satisfaction with care from doctors, nurses, and other staff. Table 4 provides detailed scores on general care.

**TABLE 4:** Experience of care with the general service.

Patients’ experience	Response		Chi-square	Probability
	Inpatients	Outpatients		
**Experience in general**				
Waiting takes a long time			30.76	< 0.001
Agree	76	44		
Neutral	68	64		
Disagree (201)	16	52		
Have access to the hospital			3.64	0.1619
Agree	89	92		
Neutral	60	61		
Disagree (202)	11	7		
Reception is welcoming			2.97	0.226
Agree	90	83		
Neutral	62	62		
Disagree	8	15		
Clean linen provided			6.16	0.0459
Agree	73	60		
Neutral	98	70		
Disagree	25	30		
**Experience specific to the environment**				
Clean environment			6.52	0.0382
Agree	75	60		
Neutral	61	65		
Disagree	24	35		
Service is good			2.17	0.3381
Agree	85	90		
Neutral	66	63		
Disagree	9	7		
Satisfied with nursing care			6.91	0.0316
Agree	83	90		
Neutral	69	63		
Disagree	8	7		
Will use the hospital in the future			4.06	0.1309
Agree	80	88		
Neutral	64	64		
Disagree	16	8		
Will recommend the hospital			3.09	0.2124
Agree	81	88		
Neutral	73	63		
Disagree	16	9		

*Source*: Adapted from Donabedian, A., 2005, ‘Evaluating the quality of medical care’, *Milbank Quarterly* 83(4), 691–729. https://doi.org/10.1111/j.1468-0009.2005.00397.x

**FIGURE 1:**
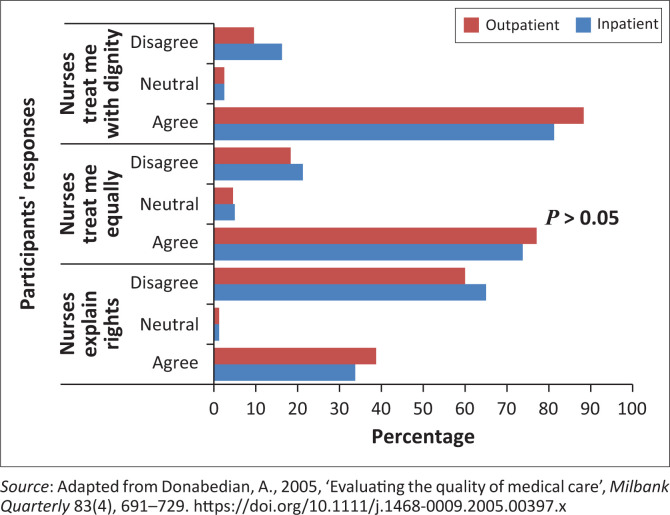
General patients’ rights application.

Figure 1 presents the association between patients’ category (inpatients, outpatients) and their satisfaction in the application of patients’ rights. Disagreement about nurses explaining patients’ rights did not differ significantly between inpatients and outpatients (*p* > 0.05), indicating rights were explained similarly to both groups. Although most respondents disagreed that their rights were explained, over 70% in both groups agreed they were treated equally and with dignity, showing no preference for either category (*p* > 0.05).

**FIGURE 2:**
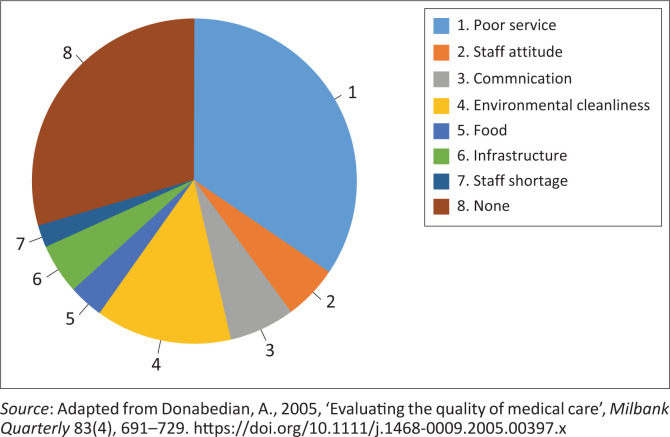
Aspects that led to patient dissatisfaction.

Figure 2 presents a summary of aspects that led to patient dissatisfaction. With reference to Figure 1, 34% respondents were dissatisfied with the service, citing dirty hospital linen and long Out Patient Department (OPD) waiting times. Several patients also noted the lack of drinking water in the OPD. Environmental hygiene ranked second (14%), with complaints about toilet conditions. Contact with staff received the lowest positive rating (7%). Infrastructure issues were mentioned by 5%, while food (3%) and staff attitude (5%) drew few complaints, which is encouraging. Staff shortages were not seen as a factor. Nearly 29% chose not to comment.

**FIGURE 3:**
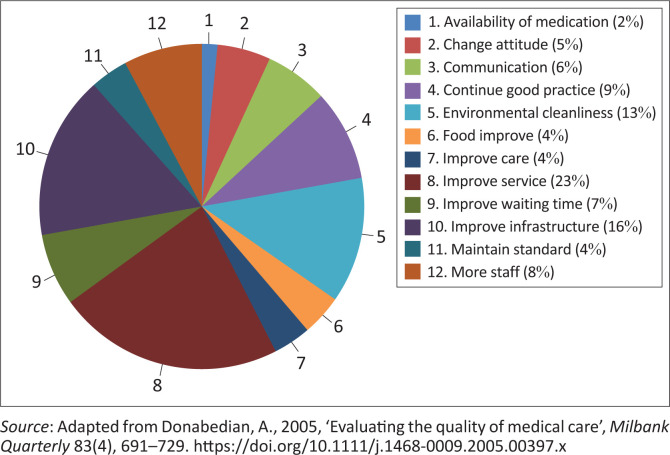
Recommendations to improve patient satisfaction.

Figure 3 presents a summary of recommendations put forth by patients to improve patient satisfaction. Respondents suggested improvements in general service (23%), infrastructure (16%), environmental hygiene (13%), staffing (8%), waiting time (7%), and communication (6%). Others recommended maintaining high standards. Patients are a key source of feedback on hospital performance, as their expectations are shaped by personal experience or information from others. Patient satisfaction for the study was evaluated based on general service, infrastructure, environmental hygiene, waiting time, and staff availability.

## Discussion

The study aimed to examine patient satisfaction during healthcare delivery at the selected district hospital. Section A covered demographic information. Applying a gender perspective can help identify factors that influence health burdens, access to health services, and responses to interventions (Mohan et al. 2025:3). Multiple factors act as barriers and facilitators for both men and women in utilising healthcare; however, generally, women show higher rates of healthcare-seeking behaviour and utilisation compared to men across all health concerns (Mohan et al. 2025:4). Women may be more prone to diseases than men and might have higher expectations during visits to healthcare facilities than men. Nonetheless, men’s underutilisation of healthcare services has been linked to societal pressures, including traditional masculinity norms and the stigma associated with seeking help (Mohan et al. 2025:4). This supports the demographic data in this study, which showed more female (67%) than male (33%) respondents (*p* < 0.001, *χ*^2^ = 37.81). Regarding age, a study by Adewole, Tumbo, and Okonta (2024:3) found that the 15–64 years age group (62.7%) in South Africa visits healthcare facilities mainly for trauma-related conditions. The 15-year-olds and below are mostly teenagers who engage in risky behaviours, whereas the 64-year-olds and above are prone to diseases as a result of low immunity. These findings are also supported by Khotib, Suprapto and Indasah (2024). The relationship between education and health is influenced by three main mediators: economic, social-psychological, and behavioural factors. Economic factors such as income and occupation affect access to medical care, making educated individuals more likely to seek treatment promptly compared to those with less education (Raghupathi & Raghupathi 2020:2). According to Tien et al. (2021), educated people have good assessment skills to evaluate the services they receive and can make informed decisions about their care. The studies thus confirm that a higher percentage of people with secondary education (59%) and tertiary education (21%) utilise healthcare facilities, compared to only 8% with no schooling and 12% with primary education. This also explains the visit patterns, where visits are higher on an occasional or monthly basis rather than weekly or daily.

Section B assessed satisfaction with care received. Communication has been identified as a variable. Effective communication is a cornerstone of quality healthcare because it helps providers bond with patients, forming therapeutic relationships that benefit patient-centred outcomes (Sharkiya 2023:5). The importance of communication in ensuring patient satisfaction is affirmed by the results and recommendations of the study. A study by Khotib et al. (2024) affirms that when healthcare professionals are kind and compassionate, the recipients of healthcare are satisfied. Communication that is patient-centred, empathetic, and clear fosters confidence, assurance, and collaborative decision-making (Omaghomi et al. 2024).

Section C focuses on general aspects and environmental issues that determine patients’ satisfaction in healthcare facilities. The cleanliness of a healthcare facility is an important determinant of an infection-free environment. Patients experience stress and fear of infection from poor environmental conditions. Poor conditions further undermine the patient’s dignity, privacy, and the timeliness of care (Parry et al. 2022). It is for this reason that several respondents of the study raised concerns about a dirty environment and other related issues, such as toilets, bathrooms, unclean water, linen, and poor infrastructure (Parry et al. 2022). Considerable efforts must be made to improve the quality of cleanliness in the patient’s environment. Thorough cleaning of healthcare facilities and disinfection reduce hospital-acquired infections. Additionally, patients must be nursed in an environment that does not pose a risk to them (Parry et al. 2022). Other aspects, such as time spent in a healthcare facility, are key. It is for this reason that the waiting time was a concern for respondents. Patients expressed unhappiness with the waiting time to see the healthcare professionals. Timely appointments and fair waiting periods help to increase overall patient satisfaction by providing efficient scheduling, thus shortening waiting times, and managers must therefore use technology to send reminders of their appointments to patients (Bell et al. 2020).

Section D highlights the recommendations shared by respondents to assist in improving the healthcare facilities. The recommendations were based on identified problems. Improvements in general service, infrastructure, environmental hygiene, staffing, waiting time, communication, and maintenance of high standards in healthcare facilities were recommended. Based on the discussion of sections A, B, and C, there is no doubt that, should the recommendations be applied, the quality of care will improve, thus improving patient satisfaction, as affirmed by several studies (Bell et al. 2020; Omaghomi et al. 2024; Parry et al. 2022; Sharkiya 2023). When patients are satisfied, they are likely to make a repeat visit to the healthcare facility because of the previous care that they received, thus confirming patient satisfaction (Khotib et al. 2024).

### Limitations

The research was conducted in one province of the country, thus making it difficult to conclude that factors leading to patient dissatisfaction in healthcare facilities around the country are similar. The findings cannot, therefore, be projected to other hospitals in the country. Moreover, the exclusion of the emergency room may have been a limitation in terms of accessing a patient in the emergency room.

### Recommendations

Further studies that focus on determining factors that lead to patient satisfaction in the country can be conducted. Monitoring and evaluation processes are to be emphasised and continuously implemented in healthcare facilities.

## Conclusion

The purpose of this study was to identify the factors that influence patient satisfaction during healthcare delivery. Several causes of patient dissatisfaction were identified, along with recommendations to address them. Much still needs to be done in healthcare facilities to ensure patient satisfaction. Therefore, it is necessary to implement the recommendations provided by service recipients. If these recommendations are effectively applied, they can enhance the quality of care, promote patient satisfaction, and reduce litigation resulting from mismanagement or ill treatment in healthcare facilities.
